# A female patient with GSD IXc developing multiple and recurrent hepatocellular carcinoma: a case report and literature review

**DOI:** 10.1038/s41439-021-00172-8

**Published:** 2021-12-08

**Authors:** Jun Kido, Hiroshi Mitsubuchi, Takehisa Watanabe, Keishin Sugawara, Hideo Sasai, Toshiyuki Fukao, Kimitoshi Nakamura

**Affiliations:** 1 Department of Pediatrics, Kumamoto University Hospital, Kumamoto, Japan; 2 Department of Pediatrics, Faculty of Life Sciences, Kumamoto University, Kumamoto, Japan; 3 Department of Neonatology, Kumamoto University Hospital, Kumamoto, Japan; 4 Department of Gastroenterology and Hepatology, Faculty of Life Sciences, Kumamoto University, Kumamoto, Japan; 5 Department of Pediatrics, Graduate School of Medicine, Gifu University, Gifu, Japan

**Keywords:** Metabolic disorders, Genetics research

## Abstract

Glycogen storage disease type IX (GSD IX), the most common form of GSD, is caused by a defect in phosphorylase kinase (PhK). We describe the case of a female patient with GSD IXc harboring a homozygous mutation in *PHKG2* (NM_000294.3; PHKG2 (c.280_282delATC (p. I94del)) definitively diagnosed using the GSD gene panel. She presented with hypoglycemia, hepatomegaly, and short stature and died of cirrhosis and recurrent multiple hepatocellular adenoma at the age of 69 years and 11 months.

Glycogen storage disease type IX (GSD IX) is one of the most common forms of GSD caused by a defect in phosphorylase kinase (PhK). This disease accounts for 25% of all GSD cases, with an estimated frequency of 1 in 100,000 individuals^1^. PhK (EC 2.7.1.38) is a serine/threonine-specific complex protein kinase consisting of four subunits: alpha (α), beta (β), gamma (γ), and delta (δ)^2^. PhK activates glycogen phosphorylase (EC 2.4.1.1), which releases glucose-1-phosphate from glycogen^3^. Patients with GSD IX have elevated transaminases and hepatomegaly, growth retardation, hypertriglyceridemia, and hypercholesterolemia. However, compared to that in patients with other types of liver GSDs, clinical manifestation in GSD IX patients can be mild, and patients may become asymptomatic as they age^4,5^. Mutations in *PHKA1*, *PHKA2*, or *PHKB* and *PHKG2* cause GSD IX. *PHKG2* encodes the hepatic isoform of the γ unit of PhK. Mutation in *PHKG2* causes GSD IXc (MIM: 613027), a rare form that manifests as hepatomegaly, hypotonia, and growth retardation in childhood. Although these symptoms improve with age, some patients develop hepatic fibrosis, cirrhosis, and liver dysfunction^6^.

A female patient was definitively diagnosed with GSD IXc at the age of 68 years using the GSD gene panel. She was diagnosed with hepatic GSD in childhood but self-discontinued clinical examination and treatment; later, she developed liver cirrhosis and hepatocellular carcinoma (HCC). Herein we present her clinical course and discuss the mechanism and treatment of HCC in GSD IXc based on her clinical course.

A 67-year-old female patient with suspected GSD was introduced to our institution to receive a definitive diagnosis while receiving conservative care therapy for liver cirrhosis and multiple HCC. She was the first child of healthy consanguineous parents. Her height was 141.9 cm (the average Japanese female height at 65–69 years is 153.3 ± 5.7 cm), and her weight was 54.7 kg. She presented with anasarca and ascites due to cirrhosis and HCC. She had no drinking habit and no history of hepatitis B or C infection. She also had no history of blood transfusion before receiving hepatectomy at the age of 56 years. Her intelligence was not impaired, and she could write advanced sentences. Hepatomegaly was first detected in this patient at 7 years; however, the diagnosis was not completed. At 12 years, she was diagnosed with GSD (type: unknown) at our institution, but she self-discontinued regular follow-up. At 30 years of age, she visited the Department of Internal Medicine because of frequent upper abdominal pain and fasting hypoglycemia symptoms, such as irritation and wooziness. In addition, she developed multiple adenomas and cirrhosis of the liver and received regular follow-up and medicine, including a gastrointestinal drug every month (Supplemental Data 1). She gradually lost the ability to work due to fatigue and hypoglycemia symptoms and lived a single life, receiving a pension under the social security system. At the age of 56 years, she underwent S7 partial hepatectomy because of HCC (Fig. 1A–E). At 63 years, she experienced multiple HCC relapse and underwent combination therapy of S4 partial hepatectomy and radiofrequency ablation (Fig. 1F). At the age of 66 years, she developed esophageal varix and underwent endoscopic variceal ligation (EVL), and she developed multiple recurrent HCCs and underwent transcatheter arterial chemoembolization (TACE) at the age of 67 years. In addition, she developed hepatic coma after rupture of the esophageal varix, which was rescued by EVL. At the age of 68 years, she developed recurrent multiple HCC and underwent a second TACE. After that, she could not undergo aggressive therapy for recurrent HCC because of impaired hepatic function and liver cirrhosis (Fig. 1G and Supplemental Data 1).

**Fig. 1:**
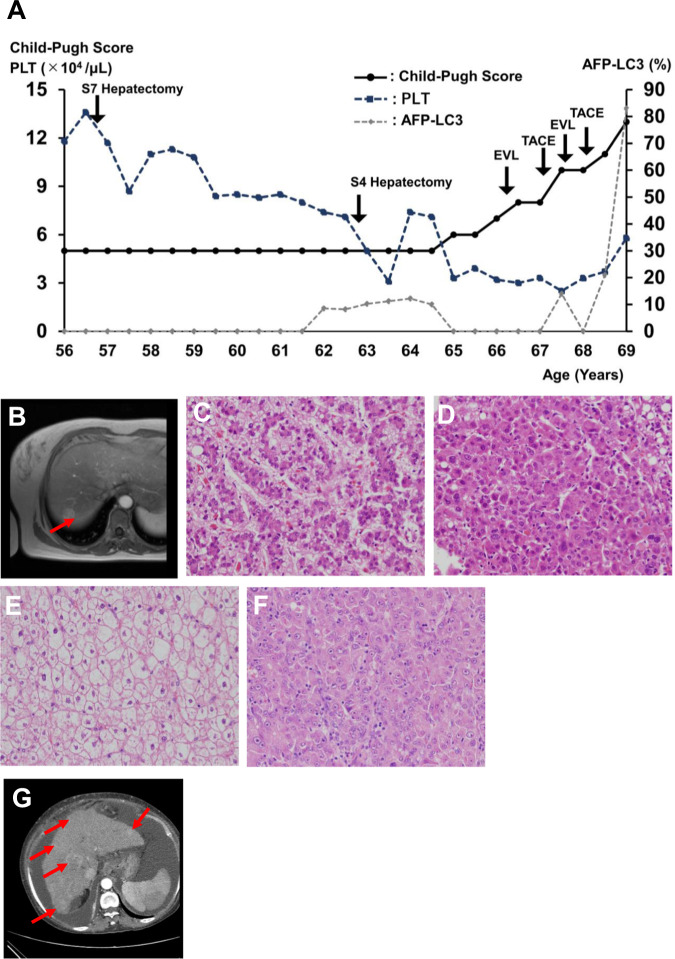
Hepatocellular carcinoma (HCC) in a female patient with GSD IXc. **A** Clinical course. Deterioration of cirrhosis and HCC progressed with age. **B** Liver MRI. T2-weighted imaging detected a 2-cm tumor in the liver (S7 section). **C**–**F** Pathological tissues. The S7 tumor exhibited characteristics of well-differentiated (**C**) and moderately differentiated (**D**) HCC combined with hepatocytes with a clear cell cytoplasm (**E**), consistent with pathology in GSD. The S4 tumor was poorly differentiated (**F**) and moderately differentiated HCC. **G** Liver CT. Multiple HCC and ascites were detected in the abdomen.

She was definitively diagnosed with GSD IXc because she harbored a homozygous c.280_282delATC (p. Ile94del) mutation in *PHKG2*. She died under palliative care at 69 years and 11 months.

Written informed consent was obtained from the patient’s parents. This study was approved by the Institutional Ethics Committee of the Faculty of Life Science, Kumamoto University.

We present a female patient with GSD IXc who developed liver cirrhosis and multiple HCC. She underwent aggressive surgical and medical treatment until the age of 68 years. However, she did not modify her diet, including consuming uncooked cornstarch for GSD, owing to her specific characteristics, including being stubborn, obsessive, and unwilling to accept others’ opinions. She was not aware of the need to follow a diet suitable for preventing hypoglycemia. To our knowledge, this is the first report of a patient with GSD IXc developing multiple HCC and receiving aggressive surgical and medical treatment. She carried a novel mutation in *PHKG2*. We have summarized the case report of GSD IXc in Table 1 (Supplemental Data 2).

**Table 1:** *PHKG2* mutations identified in the patients with GSD IXc.

Patient (sex)	Ethnicity	Age (diagnosis/current)	Variants Allele 1/Allele 2	Symptoms	Ref.
1 (M)	Caucasian	12 m/16 y	c.22G>T (p.E8*)/c.158_160delAGA (p.K53del)	Hepatomegaly, prominent cheeks, hypotonia, liver fibrosis, hypoglycemia, ketosis, elevated ASL/ALT, mild gross motor delay	^18^
2 (M)	Chinese	1 y 5 m/1 y 5 m	c.79_88delinsTCTGGTCG (p.K27Sfs*33)/c.166G>T (p.E56*)	Severe hepatomegaly, hypoglycemia, elevated ASL/ALT, hypertriglyceridemia, growth retardation	^21^
3 (F)	Caucasian	16 m/6 y	c.96-11G>A/c.247C>T (p.Q83*)	Hepatomegaly, liver fibrosis, hypoglycemia, ketosis, elevated ASL/ALT, normal growth	^18^
4 (M)	Caucasian	12 m/5 y	c.96-11G>A/no mutation identified	Hepatomegaly, elevated ASL/ALT, growth delayed, mild speech delay, significant mid-foot pronation, slight hind-foot valgus, slight genu valgus requiring orthoses, left hydronephrosis	^18^
5 (F)	Pakistani	1.5 y/2.5 y	c.107C>T (p.S36F) (homo)	Hypoglycemic seizure, hepatomegaly, elevated ASL/ALT, improved by dietary	^22^
6 (F)^a^	Pakistani	13 y/14 y	c.107C>T (p.S36F)/c.226C>T (p.R76*)	Tonic clonic seizures (since 8 m), Elevated ASL/ALT, Liver glycogenosis with mild fibrosis	^22^
7 (M)^a^		10 y/11 y		Tonic clonic seizures (since 15 m), elevated ASL/ALT, liver glycogenosis with mild fibrosis	
8 (F)	NA	6 m/18 y	c.121T>C (p.C41R)/c.643G>A (p.D215N)	Hepatomegaly, elevated ASL/ALT	^16^
9 (F)	Saudi Arabian	11 m/8 y	c.130C>T (p.R44*) (homo)	Hepatomegaly, growth retardation, elevated ASL/ALT, liver cirrhosis	^6^
10 (F)	Pakistani	2 y 4 m/NA	c.144delC (p.H48Qfs*5) (homo)	Hepatomegaly, hypoglycemia, muscle weakness, fatigue, delayed puberty	^23^
11 (M)	Pakistani	6 m/13 m	c.247C>T (p.Q83*) (homo)	Hypoglycemic seizure, hepatomegaly, improved by dietary	^22^
12 (M)	NA	6 m/17 y	c.256G>A (p.G86S)/c.925C>T (p.R309W)	Hepatomegaly, elevated ASL/ALT, failure to thrive	^16^
13 (F)	Norwegian	5 m/18 y	c.265_266insC (p.H89Pfs*13) (homo)	Hepatomegaly, muscular hypotonia, growth retardation, hypoglycemia	^24^
14 (F)	Norwegian	4 m/7.5 y	c.265_266insC (p.H89Pfs*13)/c.900G>A (p.W300*)	Hepatomegaly, hypoglycemia, elevated ASL/ALT, mild liver fibrosis, markedly retarded growth	^8^
15 (M)	Algerian	8 m/NA	c.272-1G>C (homo)	Hypoglycemia, growth delay, distal amyotrophia, elevated ASL/ALT, cirrhosis, portal fibrosis	^9^
16 (F)	Japanese	15 y/26 y	c.277delC (p.L93Sfs*17)	Hepatocellular adenoma, liver cirrhosis	^6^
17 (F)	Japanese	NA/died at 70 y	c.280_282delATC (p.I94del) (homo)	Hepatomegaly, elevated ASL/ALT, growth impairment, liver cirrhosis, ascites, multiple hepatocellular carcinoma	This study
18 (F)	Pakistani	15 m/NA	c.317T>G (p.V106E) (homo)	Hepatomegaly, growth retardation, severe liver fibrosis, elevated ALT and triglycerides, proliferation of bile ducts	^24^
19 (F)	Turkish	3.5 y/9.5 y	c.326+1G>A (homo)	Hepatomegaly, splenomegaly, liver fibrosis, cirrhosis	^25^
20 (M)	Pakistani	2 y/NA	c.431T>C (p.L144P) (homo)	Hepatomegaly, hypoglycemia, muscle weakness, fatigue, hyperlactic acidemia, autoimmune type 1 diabetes	^23^
21 (M)	English	7 m/3 y	c.433C>T (p.H145Y)/c.677T>G (p.L226R)	Poor growth, muscle wasting, hepatomegaly, elevated ASL/ALT and triglycerides	^4^
22 (M)	Pakistani	2.5 y/2.5 y	c.454C>T (p.R152*) (homo)	Elevated ASL/ALT, hepato-splenomegaly	^22^
23 (F)	Chinese	4 m/18 y	c.469G>A (p.E157K) (homo)	Hepatomegaly, elevated ASL/ALT, progressive splenomegaly and portal hypertension (starting from 7 y)	^8^
24 (F)	Chinese	3 y/3 y		Severe hepatomegaly, hypoglycemia, elevated ASL/ALT, hypertriglyceridemia, growth retardation	^21^
25 (F)	Chinese	1 y 6 m/1 y 6 m	c.469G>A (p.E157K)/c.553C>T (p.R185*)	Severe hepatomegaly, hypoglycemia, elevated ASL/ALT, hypertriglyceridemia, growth retardation	
26 (M)	Chinese	1y8m/1y8m	c.469G>A (p.E157K)/c.761delC (p.E256Sfs*12)	Severe hepatomegaly, hypoglycemia, elevated ASL/ALT, hypertriglyceridemia, growth retardation	
27 (M)	Chinese	2y1m/2y1m	c.469G>A (p.E157K)/c.835C>T (p.R279C)	Severe hepatomegaly, hypoglycemia, elevated ASL/ALT, hypertriglyceridemia, growth retardation	
28 (M)	NA	5 m/NA	c.502C>T (p.R168*)/c.859C>T (p.Q287*)	Hypoglycemia, elevated ASL/ALT, cirrhosis	^9^
29 (M)	Chinese	2 y/3 y	c.553C>T (p.R185*) (homo)	Hepatomegaly, elevated ASL/ALT, high total bile acid	^26^
30 (M)	Pakistani	2 y/2.5 y		Progressive abdominal distention, hepatomegaly, elevated ASL/ALT	^22^
31 (F)^a^	Pakistani	9 m/10 y	c.557-3C>G (homo)	Hypoglycemic seizure, elevated serum triglycerides, improved by dietary	^22^
32 (M)^a^		3 m/7 y		Hepatomegaly, improved by dietary	
33 (F)	French	7 m/5 y	c.566G>A (p.G189E) (homo)	Hepatomegaly, growth retardation, mild muscule hypotonia, elevated ASL/ALT and triglycerides	^24^
34 (F)	Norwegian	9 m/8 y	c.643G>A (p.D215N) (homo)	Hepatomegaly, hypoglycemia, elevated ASL/ALT, gastric tube feeding (from 1 y 3 m to date (8 y))	^8^
35 (M)	Caucasian	44 m/12 y	c.647+5G>T (homo)	Hypoglycemic seizure, hepatomegaly, liver fibrosis, elevated ASL/ALT, mild muscle weakness, mild delay in walking, normal growth but improved on therapy	^18^
36 (F)^a^	Saudi Arabian	9 m/>2.5 y	c.659G>A (p.G220E) (homo)	30-week gestation, 1.6 kg birth weight, delayed developmental motor milestones, hypoglycemia, hepatomegaly, liver fibrosis, elevated ASL/ALT, cirrhosis, hypocalcemia	^17^
37 (F)^a^		8 m/2.5 y		Full-term normal delivery, motor delay, hypoglycemia, hepatomegaly, liver fibrosis, elevated ASL/ALT	
38 (F)		9 m/2 y		Hypoglycemia, hepatomegaly, liver fibrosis, elevated ASL/ALT	
39 (M)	Comoran	Early childhood/>21 y	c.802_805delATCT (p.I268Pfs*12) (homo)	Hypoglycemia, cirrhosis, muscular defect with distal amyotrophia, received a liver transplantation at age 20 years	^9^
40 (F)	Caucasian	2 y/9 y	c.900G>A (p.W300*)/c.1073A>G (p.Y358C)	Full-term normal delivery, 3 kg birth weight, hepatomegaly, liver fibrosis, elevated ASL/ALT, mild muscle weakness, normal growth but improved on therapy	^18^
41 (M)^a^	Pakistani	3 y/11 y	c.958C>T (p.R320*) (homo)	Hypoglycemia, elevated ASL/ALT, noticed hepatomegaly at 2.5 y, but remained well by dietary	^13^
42 (M)^a^		3 m/7.5 y		Hepatomegaly, elevated ASL/ALT	
43 (M)	Jordonian	14 m/>6 y	c.1034C>G (p.S345*) (homo)	Hepatomegaly, elevated ASL/ALT	^27^

*NA* not available.

^a^Siblings.

The clinical outcome in 30 patients with GSD IXc was collected in an earlier study^7^. Hepatomegaly, fasting hypoglycemia, fasting ketosis, hypertriglyceridemia, hypercholesterolemia, growth delay, developmental delay, and elevated transaminase were present in 100% (30/30), 94.7% (18/19), 100% (6/6), 94.4% (17/18), 45.4% (5/11), 70.8% (17/24), 50% (10/20), and 100% (22/22) of patients with GSD IXc, respectively. Eighty percent (24/30) of patients underwent a liver biopsy. According to pathology reports, 95.8% of patients (23/24) presented with either fibrosis (mild, moderate, or severe) or cirrhosis. Three patients developed a hepatic adenoma. Only one patient developed HCC at the age of 27 years; this patient is awaiting liver transplantation.

There is no consensus on effective treatment for GSD IXc. Nasogastric tube feeding, uncooked cornstarch (0.3–2.0 g/kg body weight (BW)/day), and a high-protein diet (3.0–4.0 g/kg BW/day) are common treatments for GSD IXc^4,8,9^. Poor metabolic control is a risk factor for the development of long-term complications such as hepatocellular adenoma in hepatic GSD^10–13^. Moreover, hepatocellular adenoma may be regressed in a well-controlled metabolic state with strict dietary therapy. Nevertheless, the effectiveness of strict diet therapy is controversial because there are few adult patients with GSD IXc who receive long-term strict diet therapy.

We believe that this patient should have received a liver transplant when she was diagnosed with HCC. We had reservations in treating her HCC, which developed at the age of 58 years, particularly with regard to choosing between aggressive invasive treatment, including hepatectomy and TACE, and conservative treatment because HCC is known to recur. Overall, it is important to control the radical metabolic state in metabolic disease.

Cirrhosis is the most important risk factor for the development of HCC, regardless of etiology^14^. Liver fibrosis and hepatocellular carcinoma occur in patients with GSD VI^15,16^ caused by a defect in glycogen phosphorylase. GSD IXc with pathogenic variants in *PHKG2* is associated with more severe clinical and biochemical abnormalities, including increased risk for liver fibrosis and cirrhosis^15,17,18^, because γ is the catalytic subunit, whereas α and β are regulatory subunits of PhK. Glycogen is a key energy store for cancer cells^19^, and glycogen turnover allows cancer cells to adapt and survive under adverse oxygen and nutrient conditions within the tumor microenvironment. Glycogen breakdown supports the pentose phosphate pathway, which generates the nucleotides required for proliferation and DNA repair, and NADPH, which is an important reducing agent for reactive oxygen species scavenging and for the synthesis of nucleotides, amino acids, and lipids^19^. Therefore, both cirrhosis and glycogen accumulation are important factors in recurrent and multiple HCC. Activated PhK β-subunit expression regulates the occurrence of HCC by inhibiting AKT/protein kinase B and signal transducer and activator of transcription 3 signaling pathway activation, independent of the glycogenolytic pathway^20^. In hepatic GSD, the formation and proliferation of HCC may be related to a mechanism independent of the glycogenolytic pathway, which should be investigated in the future.

The specific characteristics of our patient might be a clinical manifestation of GSD IXc. We should have realized this possibility earlier and administered appropriate counseling.

In conclusion, we present the case of a patient with GSD IXc who developed liver cirrhosis and multiple HCC. This case shows the long-term clinical course in GSD IXc. Liver cirrhosis and HCC were important complications in this long-term surviving patient with GSD IXc. However, there is no established effective therapy for GSD IXc, and it is not clear to what extent HCC should be aggressively treated with invasive treatment. Knowledge of clinical outcomes in more patients with GSD IXc is essential to design a treatment course for GSD IXc.

## Supplementary information

Supplemental data 1

Supplemental data 2

## Data Availability

The relevant data from this Data Report are hosted at the Human Genome Variation Database at 10.6084/m9.figshare.hgv.3103.
