# Supplementary material for: A female patient with GSD IXc developing multiple and recurrent hepatocellular carcinoma: a case report and literature review
Source: Hum Genome Var. 2021 Dec 8;8:45. doi: 10.1038/s41439-021-00172-8 (PMC8651689; doi:10.1038/s41439-021-00172-8)
Supplement: Supplementary file 1 — Supplemental data 1 [file 41439_2021_172_MOESM1_ESM.docx]

|  | 51 years 2 months^a^ | 69 years 3 months (Age) | Control reference value |
| --- | --- | --- | --- |
| Total protein (g/dL) | 7.9 | 6.9 | 6.6-8.1 |
| Albumin (g/dL) | 4.3 | 2.0 | 4.1-5.1 |
| Na (mmol/L) | NA | 138 | 138-145 |
| K (mmol/L) | NA | 3.8 | 3.6-4.8 |
| Cl (mmol/L) | NA | 110 | 101-108 |
| Ca (mg/dL) | NA | 8.1 | 8.8-10.1 |
| Uric acid (mg/dL) | 5.1 | 3.6 | 2.6-5.5 |
| Blood urea nitrogen (mg/dL) | NA | 9.1 | 8.0-20.0 |
| Creatinine (mg/dL) | NA | 0.48 | 0.46-0.79 |
| Total bilirubin (mg/dL) | NA | 3.1 | 0.4-1.5 |
| Direct bilirubin (mg/dL) | NA | 1.0 | 0-0.2 |
| NH_3_ (μg/dL) | NA | 106 | 15-65 |
| Aspartate aminotransferase (U/L) | 36 | 59 | 13-30 |
| Alanine aminotransferase (U/L) | 15 | 16 | 7.0-23 |
| Lactate dehydrogenase (U/L) | 154 | 263 | 124-222 |
| **γ**-glutamyl transpeptidase (U/L) | 58 | 57 | 9-32 |
| Alkaline Phosphatase (U/L) | 203 | 426 | 106-322 |
| Cholinesterase (U/L) | 104 | 56 | 201-421 |
| Amylase (U/L) | NA | 166 | 44-132 |
| Creatine kinase (U/L) | 51 | 65 | 41-153 |
| C-reactive protein (mg/dL) | NA | 2.08 | 0.0-0.14 |
| Total cholesterol (mg/dL) | 193 | 151 | 142-248 |
| Triglyceride (mg/dL) | 103 | 55 | 30-117 |
| Blood sugar (mg/dL) | 80 | 127 | 73-109 |
| HbA1c (%) | NA | 3.8 | 4.9-6.0 |
| Lactate (mg/dL) | 14.1 | N. A | 3.2-10.9 |
| Pyruvate (mg/dL) | 1.2 | N. A | 0.23-0.74 |
| Total ketone body (μmol/L) | 112.0 | N. A | 26-122 |
| Acetoacetic acid (μmol/L) | 56.8 | N. A | 13-69 |
| **3**-hydroxybutyric acid (μmol/L) | 54.8 | N. A | 0-72 |
| Ferritin (ng/mL) | NA | 21.1 | 3-132 |
| α-fetoprotein (ng/mL) | 3.5 | 38.4 | < 7.0 |
| α-fetoprotein -LC3 (%) | NA | 83 | <10 |
| Protein induced by vitamin K absence or antagonist-II (mAU/mL) | NA | 995 | < 40 |
| Carcinoembryonic antigen (ng/mL) | NA | 6.1 | < 3.4 |
| White blood cell (×10^3^/μL) | 5.1 | 2.7 | 3.3-8.6 |
| Red blood cell (×10^6^/μL) | 3.69 | 2.38 | 3.86-4.92 |
| Hemoglobin (g/dL) | 11.7 | 8.1 | 11.6-14.8 |
| Platelet (×10^3^/μL) | 168 | 58 | 158-348 |
| Prothrombin time (%) | NA | 46 | 80-120 |
| Prothrombin time -INR | NA | 1.59 | - |
| Activated partial thromboplastin time (sec) | NA | 39.8 | 24.0-39.0 |
| Activated partial thromboplastin time (%) | NA | 54 | - |

Supplemental data 1. The laboratory data in this case

NA: not available. The blood sample was acquired 2 hours after taking lunch.

a: The height at 51 years was 145.0 cm (The average Japanease female height at 50-59 years: 153.0 cm).
