# Supplementary material for: A female patient with GSD IXc developing multiple and recurrent hepatocellular carcinoma: a case report and literature review
Source: Hum Genome Var. 2021 Dec 8;8:45. doi: 10.1038/s41439-021-00172-8 (PMC8651689; doi:10.1038/s41439-021-00172-8)
Supplement: Supplementary file 2 — Supplemental data 2 [file 41439_2021_172_MOESM2_ESM.docx]

Supplemental data 2. *PHKG2* mutations identified in the patients with GSD IXc

| No. | Nucleic acid | Amino acid | ClinVar | PolyPhen-2 (Score) | References |
| --- | --- | --- | --- | --- | --- |
| 1 | c.22G>T | p.E8* | Pathogenic | - | 18) |
| 2 | c.79_88delinsTCTGGTCG | p.K27Sfs*33 | - | - | g) |
| 3 | c.96-11G>A | - | Likely pathogenic | - | 18) |
| 4 | c.107C>T | p.S36F | - | Probably damaging (1.000) | f) |
| 5 | c.121T>C | p.C41R | - | Probably damaging (1.000) | 16) |
| 6 | c.130C>T | p.R44* | Pathogenic | - | 6) |
| 7 | c.144delC | p.H48Qfs*5 | - | - | a) |
| 8 | c.158_160delAGA | p.K53del | Uncertain significance | - | 18) |
| 9 | c.166G>T | p.E56* | - | - | g) |
| 10 | c.226C>T | p.R76* | - | - | f) |
| 11 | c.247C>T | p.Q83* | - | - | 18) |
| 12 | c.256G>A | p.G86S | Benign | Possibly damaging (0.727) | 16) |
| 13 | c.265_266insC | p.H89Pfs*13 | - | - | 8), d) |
| 14 | c.272-1G>C | - | - | - | 9) |
| 15 | c.277delC | p.L93Sfs*17 | Pathogenic | - | 6) |
| 16 | c.280_282delATC | p.I94del | - | - | This study |
| 17 | c.317T>G | p.V106E | - | Probably damaging (0.999) | d) |
| 18 | c.326+1G>A | - | - | - | e) |
| 19 | c.431T>C | p.L144P | Uncertain significance | Probably damaging (1.000) | a) |
| 20 | c.433C>T | p.H145Y | Pathogenic | Probably damaging (0.985) | 4) |
| 21 | c.454C>T | p.R152* | Likely pathogenic | - | f) |
| 22 | c.469G>A | p.E157K | Uncertain significance | Probably damaging (0.997) | 8), 16), g) |
| 23 | c.502C>T | p.R168* | - | - | 9) |
| 24 | c.553C>T | p.R185* | Pathogenic | - | c) |
| 25 | c.557-3C>G | - | - | - | f) |
| 26 | c.566G>A | p.G189E | Pathogenic | Probably damaging (1.000) | d) |
| 27 | c.643G>A | p.D215N | Pathogenic | Probably damaging (1.000) | 8), d) |
| 28 | c.647+5G>T | - | - | - | 18) |
| 29 | c.659G>A | p.G220E | - | Probably damaging (1.000) | 17) |
| 30 | c.677T>G | p.L226R | Pathogenic | Probably damaging (1.000) | 4) |
| 31 | c.761delC | p.E256Sfs*12 | - | - | g) |
| 32 | c.802_805delATCT | p.I268Pfs*12 | - | - | 9) |
| 33 | c.835C>T | p.R279C | - | Probably damaging (1.000) | g) |
| 34 | c.859C>T | p.Q287* | - | - | 9) |
| 35 | c.900G>A | p.W300* | - | - | 2), 8) |
| 36 | c.925C>T | p.R309W | - | Probably damaging (1.000) | 16) |
| 37 | c.958C>T | p.R320* | Uncertain significance | - | f) |
| 38 | c.1034C>G | p.S345* | - | - | b) |
| 39 | c.1073A>G | p.Y358C | - | Probably damaging (1.000) | 18) |

References

a. Beauchamp NJ, et al. (2007) Glycogen storage disease type IX: High variability in clinical phenotype. Mol Genet Metab 92: 88-99.

b. Fahiminiya S, et al. (2014) Whole exome sequencing unravels disease-causing genes in consanguineous families in Qatar. Clin Genet 86: 134-141.

c. Li C, et al. (2018) *PHKG2* mutation spectrum in glycogen storage disease type IXc: a case report and review of the literature. J Pediatr Endocrinol Metab 31: 331-338.

d. Michele AJ, et al. (1996) Mutations in the testis/liver isoform of the phosphorylase kinase gamma subunit (*PHKG2*) cause autosomal liver glycogenosis in the gsd rat and in humans. Nat Genet 14: 337-340.

e. van Beurden EACM, et al. (1997) Autosomal Recessive Liver Phosphorylase Kinase Deficiency Caused by a Novel Splice-Site Mutation in the Gene Encoding the Liver Gamma Subunit (*PHKG2*). Biochem Biophys Res Commun 236: 544-548.

f. Waheed N, et al. (2020) Variability of clinical and biochemical phenotype in liver phosphorylase kinase deficiency with variants in the phosphorylase kinase (PHKG2) gene. J Pediatr Endocrinol Metab 33: 1117-1123.

g. Zhou D, et al. (2017) Clinical features and PHKG2 gene mutation analysis of 5 Chinese patients with glycogen storage disease IXc. J Clin Pediatr 35: 609-612.
